# Influencing Factors on Airflow and Pollutant Dispersion around Buildings under the Combined Effect of Wind and Buoyancy—A Review

**DOI:** 10.3390/ijerph191912895

**Published:** 2022-10-08

**Authors:** Mei Wu, Guangwei Zhang, Liping Wang, Xiaoping Liu, Zhengwei Wu

**Affiliations:** 1 School of Civil Engineering, Hefei University of Technology, Hefei 230009, China; 2 School of Nuclear Science and Technology, University of Science and Technology of China, Hefei 230026, China

**Keywords:** wind tunnel measurements, computational fluid dynamics, thermal buoyancy, combined effect, pollutant dispersion

## Abstract

With the rapid growth of populations worldwide, air quality has become an increasingly important issue related to the health and safety of city inhabitants. There are quite a few factors that contribute to urban air pollution; the majority of studies examining the issue are concerned with environmental conditions, building geometries, source characteristics and other factors and have used a variety of approaches, from theoretical modelling to experimental measurements and numerical simulations. Among the environmental conditions, solar-radiation-induced buoyancy plays an important role in realistic conditions. The thermal conditions of the ground and building façades directly affect the wind field and pollutant dispersion patterns in the microclimate. The coupling effect of wind and buoyancy on the urban environment are currently hot and attractive research topics. Extensive studies have been devoted to this field, some focused on the street canyon scale, and have found that thermal effects do not significantly affect the main airflow structure in the interior of the street canyon but strongly affect the wind velocity and pollutant concentration at the pedestrian level. Others revealed that the pollutant dispersion routes can be obviously different under various Richardson numbers at the scale of the isolated building. The purpose of this review is therefore to systematically articulate the approaches and research outcomes under the combined effect of wind and buoyancy from the street canyon scale to an isolated building, which should provide some insights into future modelling directions in environmental studies.

## 1. Introduction

Along with the rapid expansion in the global population, environmental contamination, especially air pollution, has become a serious problem. With the development of the economy and people’s awareness of environmental protection, people are paying increasing attention to indoor and outdoor air quality [1,2,3,4,5,6,7,8,9,10,11,12]. Air pollution is bounded by the envelope of buildings and can be divided into outdoor air pollution and indoor air pollution. The primary outdoor emission sources mainly include industrial waste gas [13,14,15], traffic emissions [16,17,18,19] and construction dust [20,21,22]. Building materials, cooking odours and indoor equipment are the main sources of indoor air pollution [23,24,25]. On the other hand, the outbreaks of SARS, MERS and COVID-19, airborne human diseases, are great threats to global environmental health [26,27,28,29,30,31,32]. Thus, establishing a healthy, comfortable and safe environment, both indoors and outdoors, is the primary purpose of modern buildings.

Pollutant dispersion from emission sources is influenced by a multitude of factors, including environmental conditions, source characteristics, building morphology and the distribution of obstacles around buildings. The effect of environmental conditions, such as wind speed [33,34,35,36,37], wind direction [38,39,40,41,42], inflow turbulence [43,44,45,46,47] and thermal stratification [48,49,50,51,52,53,54,55] on airflow around buildings has attracted the attention of many researchers in recent years. The thermal action on the external wall caused by environmental heat sources such as solar radiation is also receiving increased emphasis as one of the major driving forces for airflow motion [56,57,58,59]. In urban environments, the buoyancy effect is mainly caused by solar radiation directly onto the external wall façade and ground during the daytime, thus heating the surrounding air. The street surface in hot summer could be heated to 64 °C due to the vertical incidence of solar radiation [57], and the maximum temperature difference between the exterior wall surface and surrounding air could reach 18 °C [60]. Under windless conditions, the downwards inertial force is often offset by the upwards buoyancy force, and the role of the buoyancy force on airflow movement should not be overlooked.

The airflow and dispersion in urban areas can be divided into four scales based on Britter and Hanna’s study [61]: regional (100 or 200 km), city scale (10 or 20 km), neighborhood scale (1 or 2 km) and street scale (100 to 200 m). Notably, for the urban airflow and pollutant dispersion driven by buoyancy effects, past studies mainly focused on the neighborhood scale [62,63] and street scale [64,65].

Both the wind speed and source location are important elements in determining the flow field and pollutant dispersion around isolated buildings under non-isothermal conditions. Generally, in the normal wind direction, a stagnation zone was observed around a bluff body at a height of 2/3 of the windward side of the building [66]. The airstream above the stagnation zone flows to the building top, and the airstream from below the stagnation zone flows to the floor. The wall heat plume drives the contaminants emitted from low-rise units to upper units, especially in high-rise buildings.

At the neighborhood and street scales, the aspect ratios (AR: building height/street width) are normally considered as one of the most significant parameters affecting the pollutant dispersion [67,68,69]. He et al. investigated the flow and vehicular passive emission exposure under aspect ratios from 1 to 6 and found that the pollutant exposures of the windward side were lower than those on the leeward side when AR = 1–4, but the trend was the opposite when AR = 5–6 [68]. By employing computational fluid dynamics (CFD) simulations, the results show that higher aspect ratios and ambient wind speeds increase the accumulation of pollutant concentrations in street canyons under all diurnal heating scenarios [69]. In addition, the thermal effect can also be responsible for producing a flow field in canyons. According to the results of the fluid experiment reported by Liu et al. [70], the airflow within the street canyon is driven entirely by the buoyancy force, whereas convection can arrive at the canyon’s upper atmosphere with the calm wind. The factors related to the thermal effect can be simply divided into thermal position and thermal intensity. During the daytime, solar radiation heats the different surfaces by different zenith angles [71,72], and the temperature difference between the ambient air and different surfaces varies with the daily temperature and sunshine duration variation in the street canyon [73].

In reality, because wind and buoyancy usually act together in both canyons and isolated buildings, pollutant dispersion driven by wind or buoyancy alone is nearly nonexistent [74]. Compared to iso-thermal flow studies, non-isothermal conditions have been relatively less studied, and reviews devoted to studying the effect of various factors on flow and dispersion under thermal effects are relatively scarce. Therefore, there is a need for a comprehensive survey of the flow field and dispersion for different influential factors under the combined effect of wind and buoyancy. This paper presents a comprehensive and systematic review of the existing literature on the flow fields and pollutant dispersion of two typical configurations under the combined effect of wind and buoyancy.

## 2. Materials and Methods

### 2.1. Criteria for Article Selection

This literature review was performed by searching Google Scholar, Science Direct and Web of Science papers in addition to publications known to the authors. We mainly focused on the articles published in the last twenty years. This review focuses on two typical building configurations (isolated buildings and street canyons). For each database, one set of keywords included “pollutant dispersion”, “thermal effect”, “building” or “street” and all the factors (all subtitles) mentioned in Section 3. On this basis, the relevant papers were integrated and summarized.

### 2.2. Wind-Buoyancy Interactions and Governing Parameters

In the real-world scenario, wind and buoyancy are always present in urban flows at the same time. The flow pattern in urban areas relates to the relative intensity between buoyancy and ambient wind, and several dimensionless parameters were suggested to indicate the relative intensity between these two forces in previous studies, including the Froude number (*Fr*) and the Richardson number (Ri) [75]. The Froude number is the reciprocal of the Richardson number, which means $F_{r}=\frac{1}{Ri}$. The Ri number definition is as follows:

(1) $$Ri=\frac{\mathrm{Gr}}{Re^{2}}$$

(2) $$Gr=\frac{g\beta H^{3}\left(T_{W}-T_{ref}\right)}{\nu^{2}}$$

(3) $$Re=\frac{u_{0}H}{\nu }$$

where *Gr* is the Grashof number, and *Re* is the Reynolds number. $T_{W}$ is the external wall surface temperature, and $T_{ref}$ is the ambient air temperature; *g* is the acceleration of gravity, β is the thermal expansion coefficient, *H* is the reference height, $u_{0}$ is the ambient wind velocity, and *ν* is the air kinematic viscosity. The Reynolds independence has been significant for scaled models in urban aerodynamics [76]. Castro and Robins [77] suggested a critical Reynolds number of 4000 based on the velocity at the height of the building, while Hoydysh et al. [78] suggested 3400 based on the free stream velocity. The critical Reynolds number 11,000 proposed by Snyder [79] is widely adopted to fulfil the dynamic similarity [80,81,82,83,84].

To compare the mechanical driving force and buoyant driving forces, the Richardson number (Ri) is defined. Normally, when the Ri number is smaller than 0.1, mechanical driving plays a key role. When the Ri number is larger than 10, the buoyant driving force dominates. However, in the case of a non-uniform temperature of the building facade or various heights of the buildings, the estimation of the Ri number could be complicated. For the idealized building configuration, the selection of building reference height is uncontroversial, but for the street canyon with various building heights, the definition of reference height is different in studies. Li et al. [85] used the average building heights of the step-down and step-up canyons to calculate the Ri number, while Zhao et al. took the lower building height as the reference height in the step-up canyon [86]. Therefore, cross comparisons between studies should be made with care in the choice of length scales and temperature difference.

It should be noted that there might be some expression features of Ri used in the studies [87,88,89,90]. In the study by Nazarian et al. [87], the bulk Richardson number (*Rib*) was used for the heated/cooled surface in the street canyon, and it was written as:

(4) $$Ri_{b}=\frac{\mathrm{gH}\left(T_{\mathrm{ref}}{-T}_{w}\right)}{T_{\mathrm{ref}}U_{\mathrm{ref}}^{2}}$$

where $U_{ref}$ is the mean wind speed at the reference height, and the temperature used in the equation is the average temperature. Obviously, the value from formula (4) is a negative number for the heating wall and a positive number for formula (1). In addition to this, Nazarian et al. [91] proposed the horizontal Richardson number (*Rih*), which differs from previous studies that incorporate the effect of the canyon aspect ratio (H/W). *Rih* can be defined by comparing the ratio of the vertical momentum and horizontal thermal forcing in the canyon and was written as:

(5) $$Ri_{h}=\frac{\partial F_{t}/\partial x}{\partial F_{m}/\partial z}=\frac{gH}{U_{b}^{2}}\frac{\left(\bar{T_{L}}-\bar{T_{W}}\right)}{T_{a}}\frac{H}{W}$$

where $\partial F_{t}/\partial x$ is the buoyancy force, and $\partial F_{m}/\partial z$ is the inertial force. Without thermal forcing, the momentum forcing (*Fm*) can be defined as $\frac{\partial \bar{u^{\prime }\omega^{\prime }}}{\partial z}$. Under a small bulk approaching wind, the thermal forcing (*Ft*) can be written as $g\frac{T_{W}-T_{L}}{T_{a}}$. *Ub* is the bulk wind speed, and $\bar{T_{L}}$ and $\bar{T_{W}}$ are the average temperatures of the leeward and windward surfaces, respectively. This formula takes into account the canyon aspect ratio and indicates different heating intensity effects. Alternatively, it should be noted that the expressions of the Richardson number were very diverse, including the gradient and flux Richardson number [92,93].

When the Richardson number is used in this study in the following section, it refers only to the Ri number, which is calculated from formula (1) to avoid any ambiguities in the analysis. Nakamura et al. [94] reported data measured within an actual urban street canyon and found that Ri ranged from 0.17 to 0.45 on a clear mid-summer afternoon. Uehara et al. [95] investigated the atmospheric stability effect on airflow characteristics using a stratified wind tunnel, and they found that the wind velocity inside the canyon was almost zero when Ri reached 0.4–0.8. The airflow pattern at the urban canyon layer had a close relationship with Ri.

## 3. Results and Discussion

### 3.1. Non-Isothermal Studies on Airflow and Pollutant Dispersion around an Isolated Building

#### 3.1.1. Effect of Approaching Wind

Field and meteorological observations indicate that the airflow in an urban area is usually gusty, with wind speed and direction varying with time [96]. It appears that the strong winds may function as an air curtain with regard to blocking floor heat mass transfer between units, and this was revealed by several studies [97,98]. In the numerical study by Gao et al., when the temperature difference between wall surfaces and ambient air was 5 K (*Tw − Tair* = 5 K), as the ambient wind speed increased from 0.5 m/s to 2.0 m/s, the plume of pollutant released from the room was forced to approach the upper window, and when the wind speed further increased to 4 m/s, the warm plume development was limited [97].

For non-isothermal studies, the variation in wind speed leads to different Ri numbers, and the results are commonly analysed using the Ri number [99,100,101]. For example, Huang et al. [100] investigated the heated leeward wall surface and ground on flow and dispersion of rooftop stack emissions around an isolated building under four free-stream velocities (1, 3, 6, 9 m/s), and they reported that when Ri ≤ 0.26, the resulting vortex flow field was similar to that obtained in isothermal cases. In the common isolated building under non-isothermal conditions, as the approaching wind speed increased, the extent of pollutants in the upwards direction was suppressed, while the lateral dispersion first increased and then decreased [98]. Table 1 summarizes the studies focusing on approaching wind speed effects under surface heated conditions. The Reynolds-averaged Navier–Stokes (RANS) approach was adopted in most numerical studies. This is the most commonly used method in computational fluid dynamics and does not require extensive resources [102,103,104]. However, there are well-known limitations of steady RANS methods; they can only supply the average wind flow field and dispersion fields and cannot capture the typical characteristic of the inherently transient behavior of wind flows around buildings. LES methods can meet the requirement and provide a more accurate prediction [105,106,107,108], which inevitably increases the time cost and computational resources [109]. On the other hand, the time-varying characteristics of wind speeds and directions and the turbulent fluctuation were not considered in the current existing non-isothermal study. This might be considered when planning for follow-up studies.

#### 3.1.2. Effect of Source Position

It has been confirmed that the location of the source has a significant influence on pollutant dispersion [111]. Over the last 10 years, a number of studies in the non-isothermal field around isolated buildings have investigated this issue. First, the predication of gasses emitted from rooftops has become a concern. Huang et al. carried out a series of experiments to simulate stack emissions in a wind tunnel, where pollutants were released from the left (upwind) or right (downwind) sides of the building rooftop with the rooftop heated to 400 °C [112]. The experimental data indicated that the distribution paths of pollutants also vary under different source locations, even for the same Ri number. The pollutants were all lifted upwards and discharged into the upper atmosphere. A similar study with left/middle/right sources on the rooftop showed similar results using CFD methods, and the vertical pollutant concentration of the left source position was higher than those of the center and right stacks [113].

Second, the transmission of gaseous pollutants released from multistory buildings at different heights has attracted much attention [98,99,101,114]. For the pollutant source located at the lower, middle and higher parts of the building under the heating condition of the external wall surface, some studies have been previously performed. Based on the results presented by Liu et al. [114], there was a strong near-surface dispersion effect of increasing windward temperature when the pollutant was emitted in the middle floor. When the source was located in the lower part of the leeward side, the concentration increased in both the upper and lower adjacent levels as the temperature of the leeward side increased, while the concentration decreased with the growth of leeward surface temperature when the pollutant was emitted from the higher floor of the leeward side. Furthermore, Liu et al. [98] found that when Ri ≤ 3.5, the vertical upwards transmission was restrained as the source was located at the windward side. The pollutant transmission was in the downwards and horizontal directions when the source was emitted from the leeward side, and the worst situation was observed in the case of Ri = 0.074. In addition, more experimental studies and varying-time inflow conditions should be performed to further probe the mode of transient transmission which varies with time.

#### 3.1.3. Effect of Thermal Intensity

Thermal intensity is the most common factor in non-isothermal studies to investigate the flow field and pollutant dispersion. The majority of non-isothermal studies around the isolated building all included this factor [114,115,116,117,118,119]. This factor can be broken down into thermal stratification and different surface heating intensities.

Thermal stratification comes in three forms (unstable, stable, neutral). Unstable thermal stratification generally occurs during daytime hours; there are significant temperature differences between urban surfaces and ambient air [60], and the near-ground atmosphere usually behaves as a stable stratification at night [120]. Some papers have reported comparisons between unstable and neutral stratifications of flow fields and pollutant concentration fields. Pavlidis et al. [121] noted that the velocity and temperature fields were almost identical for neutral and unstable conditions by using the PALM model. The pollutant concentration fields were studied by Masoumi-Verki et al. [122], and the results obtained revealed that unstable thermal stratification conditions led to an increase in turbulent kinetic energy (TKE), which increased concentration fluctuations and thus led to a decrease in pollutant concentrations. Farzad et al. [123] examined the mechanisms of turbulent transport and inverse gradients by employing LES methods under three different thermal stratification conditions around a high-rise building.

During the day, solar radiation heats different surfaces by different zenith angles. Depending on the latitude and time of day, the building ground, windward side, leeward side and roof could be heated. Urban flows at different thermal intensities and different heated surfaces have been studied previously. Table 2 summarizes the experimental and CFD studies with the buoyancy effect concerning thermal intensity and thermal position. Research methods, turbulence models, heated surface, heated intensity, Ri number and data availability are all included in Table 2.

The majority of studies were completed on a reduced scale, and the Reynolds number (*Re*) was lower than the full-scale model condition. Although Snyder [124] warned that “tests to establish Reynolds number independence should be an integral part of any model study”, this critical Reynolds number has been applied to non-isothermal flows without questioning its validity. The results of the scaled experiment and full-scale measurement with the same Ri number indicate that the buoyancy effect is only outstanding under reduced-scale conditions [125]. Thus, the accuracy of the Reynolds number independent criterion under non-isothermal conditions remains questionable, and it is worth exploring and investigating whether the results obtained from scaled models can be generalized to all full-scale models.

#### 3.1.4. Other Factors

Aside from the three aforementioned factors, other factors could also affect the non-isothermal flow field. For example, window type is easily understood [110,126]. Wang quantified the ventilation rates through various typical window types and found that the ventilation rates and temperature distributions inside the building varied depending on the type of window, even though the areas where the windows open were almost identical [126]. Furthermore, open window or close window mode [99], thermal position [98,99,114] and building height [119] effects were taken into consideration in different articles.

### 3.2. Non-Isothermal Studies on Airflow and Pollutant Dispersion in Street Canyons

#### 3.2.1. Effect of Approaching Wind

Similar to an isolated building scenario, the inflow wind condition also has a significant influence on the characteristics of the flow and pollutant dispersion in street canyons [127]. As the study of pollutant dispersion issues progresses in urban areas, more and more studies have probed the associations of inflow conditions and airflow characteristics in street canyons [81,94,128,129]. There are many studies on inflow conditions under non-isothermal conditions. This section will primarily discuss the wind speed effect, while the time-varying inflows and wind direction are also summarized.

**Figure 1:**
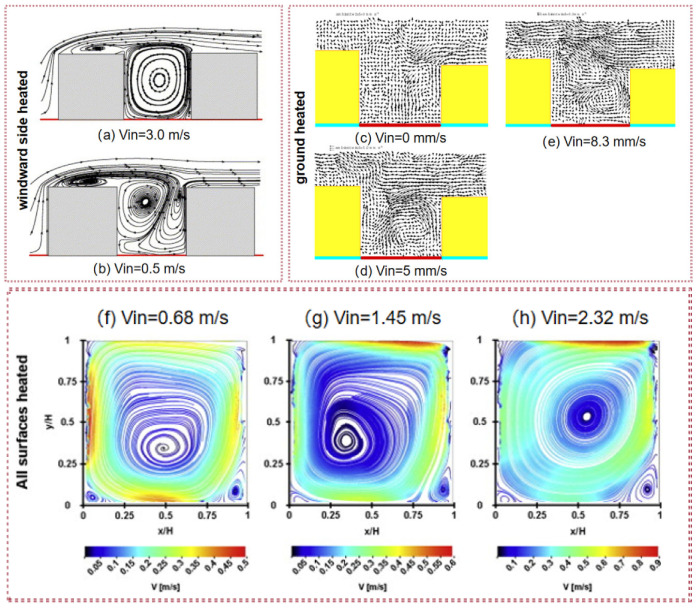
Airflow profiles at different wind speeds: (**a**,**b**) CFD results with the windward side heated. Adapted with permission from Ref. [130]. 2005, Elsevier; (**c**–**e**) water tank experiment results with the ground heated. Adapted with permission from Ref. [70]. 2003, Springer Nature; (**f**–**h**) wind tunnel experiment results with all surfaces heated. Adapted with permission from Ref. [131]. 2013, Elsevier.

For a 2D street canyon with AR = 1, if the inflow wind speed Vin = 0.5–1.0 m/s, the street canyon flow structure consisted of two parallel vortices as the ground was heated, and when the wind speed increased to 2.0–3.0 m/s, there was only one single vortex. One single vortex was observed within the windward wall that was heated, with a wind speed of 3.0 m/s, and the wind speed deceased to 0.5–2.0 m/s; the flow regime changed to two counter-rotating vortices [130]. For an asymmetric street canyon with AR = 0.83, Liu et al. studied the effect of ambient wind speed on the flow based on the particle image visualization (PIV) technique when the ground was heated in a water tank experiment [70]. The results show that the center of the vortex becomes closer to the leeward side and weakens the small circulation with increasing towing speed, as shown in Figure 1c–e. The same conclusion was found in the study of Allegrini et al. [131], who used PIV to investigate the flow field inside a canyon with AR = 1 by wind tunnel experiments. When all surfaces were heated, the main vortex was again strengthened by buoyancy for all velocities.

In addition to the airflow characteristics, the inflow wind speed also affects the air change rate inside the canyon. Wang et al. employed CFD methods to quantify the air change rate in a regular street canyon with AR = 0.75. When the inflow wind speed ranged from 0.5 m/s to 1.0 m/s, leeward and ground heating improved the air change rates in the canyon, and when the inflow wind speed was above 2.0 m/s, the thermal effect did not influence the air change rate inside the canyons, regardless of which surface was heated. [132].

Then, we will note the influence of the inflow wind speed on contamination exposed in street canyons. According to the experimental data obtained in a wind tunnel of Tongji University, Shanghai, by Cui et al. [133], when the tracer gas was located on a low-rise building rooftop with the ground heated, as the inflow wind speed increased (0.4–0.8 m/s), there was a rapid dispersion of pollutants and decreased concentrations along the street vertical center. Moreover, Xie et al. [134] noted that a higher pollutant concentration appeared on the windward side than on the leeward side as the wind velocity decreased from 2.0 m/s to 1.0 m/s in a symmetrical street with the ground heated as the source located at the street center.

Several indicators have also been previously used in non-isothermal studies to estimate the effect of wind speed, such as temperature distribution [133,135,136,137,138], turbulent kinetic energy distribution [131,137,138,139,140,141] and convective heat transfer coefficient [142]. These studies show the possibility of using a simple profile to estimate the wind speed effect with the surface heated. For street canyon environments, the majority of studies on the flow field were performed for the incident wind aligned with the canyon. Different angles of wind produce various velocity distributions and surface drag. The building roof temperature at a 45° incident wind angle was about 2.5 K lower than the incident wind at 0° and 90° angles, and the incident wind angle has no significant effect on the ground temperature [143].

#### 3.2.2. Effect of Thermal Position

Thermal position is another critical parameter affecting the flow structure in the canyons and thus affects the concentration of pollutants within the canyon. Once the sun begins to rise and move, different surfaces of the building are heated depending on different times of the day. There are no surfaces that are heated with the cloudy days or strong winds with little temperature difference, and all surfaces are heated at night by the urban heat island effect.

For a two-dimensional (2D) street canyon with AR = 1, ground heating (GH), leeward surface heating (LH) and all surface heating (AH) significantly enhance the primary clockwise vortex and improve pollutant dispersion, while windward surface heating (WH) and no surface heating (NH) do not [144]. This was also confirmed in the study by Hang et al., who extended this conclusion to street canyons with ARs ranging from 0.5 to 0.67. Furthermore, a street canyon with AR = 2 was also investigated, and they found that the conditions of ground and leeward surface heating can slightly enhance the pollutant dilution capacity and turbulence flow. Next, the AR was further scaled up to three, and windward surface heating, ground heating and all surface heating significantly increased the dispersion of pollutants near the ground [145].

For a three-dimensional (3D) street canyon, Witri et al. [146] observed an overall concentration reduction in all thermal conditions with an aspect ratio of s one, except for the windward surface heating condition, and this special result was attributed to the reduction in the overall wind speed in the street canyon, which led to pollutant accumulation. This finding is similar to those reported in studies in 2D street canyons [144,147].

Table 3 summarizes the studies concerning the thermal position effect under non-isothermal conditions. These studies present a wide variety of flow features inside the canyon with a wide range of Ri numbers. Until now, evidence to support multiple flow structures in 3D street canyons with different aspect ratios has been lacking. 3D simulation is much more expensive than 2D simulation in terms of computational cost for the same grid resolution, even up to 100–1000 times. Therefore, few studies have focused on the thermal position effect on the flow field and pollutant dispersion in 3D street canyons and have not considered the wide range of aspect ratios. Additionally, it is difficult to characterize the flow pattern in 3D, as the vortices rotate in multiple planes [148]. In summary, studies on thermal position with the buoyancy effect using 3D simulations or experimental methods in urban street canyons are still limited.

#### 3.2.3. Effect of Thermal Intensity

Thus far, the commonly used methods for creating various thermal intensities can be roughly divided into three categories: (1) thermal stability, including stable, neutral and unstable conditions [154]. In the stable stratification situation, the ground is usually maintained at a constant temperature while the recirculating air is heated (T_W_ > T_ref_). In the case of unstable stratification, the ground is heated, and the recirculating air is maintained at a lower temperature (T_W_ < T_ref_) [155]. As its name implies, the neutral condition means T_W_ = T_ref_. (2) Different uniform temperatures are imposed on a particular surface, leaving a temperature difference between ambient air and the target heated surface. (3) We simulate the solar radiation intensity at the local solar time (LST) and create non-uniform heating surfaces. Hence, these three groups are used to categorize the following studies and summarize the thermal intensity effect on airflow and pollutant dispersion in the street canyon.

As early as 2000, Uehara et al. [95] investigated the atmospheric stability effect on the airflow inside a street canyon using a stratified wind tunnel and found that as the stratification became strong, the vortices that formed in the canyon became weaker, while the greater the instability was, the stronger the vortices became. The vortex intensity was weakest under neutral conditions [156]. Cheng studied pollutant dispersion in 2D urban street canyons in neutral, unstable, and stable thermal stratifications using the LES method by OpenFOAM. The simulation results indicate that in the stable stratification situation, a stagnant air layer was formed at ground level that trapped the pollutant, leading to a severe accumulation of the pollutant. Under neutral and unstable stratification situations, the pollutants were mixed well and were distributed more evenly in street canyons. As stability decreases, the retention time of pollutants in the street canyon increases [48]. Going one step further, Cai et al. [155] established the correlations between thermal stabilities and quantitative indicators based on the CFD method. Quantitative indicators include the air change rate, pollutant removal rate, heat removal rate, heat transfer coefficient and pollutant transfer coefficient.

Nakamura and Oke [94] measured the temperature inside a street canyon with AR = 1.06 and a sky view factor of 0.43, and they discovered that the temperature difference between the building external surface and ambient air can reach 12–14 K. Based on these analyses, numerous studies have been undertaken utilizing uniform surface heating scenarios. Kim and Baik. [73] identified five flow regimes according to the simulation results of 16 different aspect ratios (0.6~3.6) and 9 heating intensities on canyon ground (0–16 K) in a 2D street canyon. When AR was very small (≤0.6) and the thermal intensity was very strong (≥10 K), as the thermal intensity increased, a thermally induced vortex (TIV) on the leeward side expanded, but a mechanically induced vortex (MIV) on the windward side contracted. When the aspect ratio was relatively small or the heating intensity was weak, an increase in the strength and size of small eddies with increasing heating intensity was also observed. With an increase in thermal intensity, the vortex increased, the center of the vortex decreased, and the upper vortex expanded inside the canyon when aspect ratios ranged from 1.2 to 2.0, and the heating intensity was above 4 K. Li et al. [157] employed the LES method to study the effect of ground heating on the pollutant concentration fields in a uniform street canyon. It has been demonstrated that increased ground heating intensity facilitates the removal of contaminants inside the canyon. This is the result of ground heating increasing updrafts, turbulence mixing and pollutant fluxes, but it also increases the temperature at pedestrian heights. Except for the ground heating, the different heating intensities of the windward/leeward surface changed the vortex structure [131]. With the increase in wall surface heating intensity, the turbulence kinetic energy was enhanced inside the canyon [152]. Table 4 denotes some articles on the effect of thermal intensity (uniform surface heating). The velocity fields, turbulent kinetic energy fields, temperature fields and pollutant concentration fields are available in various studies.

In reality, the heating of a wall or ground surface induced by solar radiation is unlikely to be uniform. Nazarian et al., initialized the corresponding time wind forcing data and temperature using the solar load model and typical meteorological year data [87]. The time of day, location, and canyon geometry determine the solar heat flow intensity and sunlit configuration. The canyon vortex strength was influenced by spatial heating patterns at the wall or ground surface that produce horizontal temperature and pressure gradients above and within the canyon.

#### 3.2.4. Effect of Canyon Geometry

**Figure 2:**
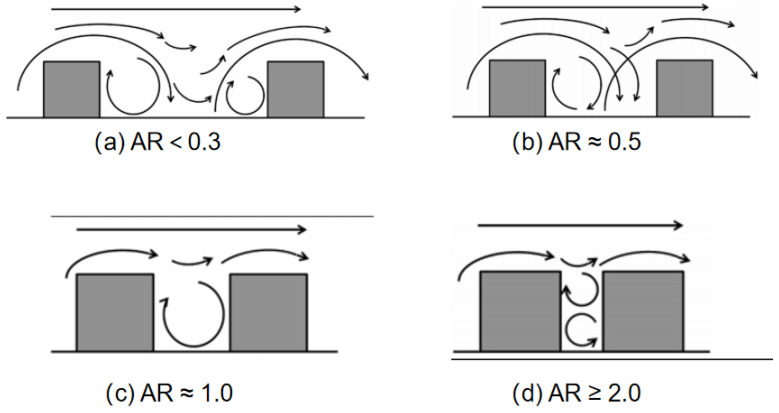
(**a**–**d**) The flow regimes in canyons for different aspect ratios under isothermal conditions. Adapted with permission from Ref. [138]. 2020, Elsevier.

The configuration of the street canyon, including architectural design, building geometry and canyon dimensions, determines the pollutant transport characteristics and airflow [150]. The aspect ratio is the most commonly used indicator to represent canyon geometry. Four kinds of flow regimes have been detected under isothermal conditions in 2D street canyons [158,159,160,161,162,163,164,165,166,167,168,169,170]. These regimes can be categorized into fully isolated roughness flow, wake interference flow, skimming flow and multivortex flow regimes in deep street canyons. In the previous literature, the descriptions of the first three flow patterns were basically consistent, as shown in Figure 2, but differed in the fourth vortex flow regime. Two contra-rotative vortexes were observed when the aspect ratio was 2.0 in the 2D wind tunnel experiment [67,169], while a single-main-vortex structure was detected when the aspect ratio was 2.7 in the full-scale street canyon [170].

When considering the thermal effect, many studies have been devoted to elucidating the aspect ratio influence on the flow field in urban street canyons [64,70,73,138,143,145,149,150]. As the ground and building surface are all heated, Mei et al. used the unsteady RANS model to simulate 6 street canyon aspect ratios between 0.5 and 3.0. In a shallower street with AR = 0.5, the flow regime was similar to that under wind-driven flows. In the canyon with AR = 1.0, two diagonally divided and counter-rotating vortices can be observed. For the street canyon with AR of 1.5 to 2.5, three vertically distributed vortices were observed, while two or three vortices were found in the canyon formed by wind. In the deeper canyon with AR = 3.0, four counter-rotating vortices can be found. The anti-clockwise vortex was divided into two vortices [149]. The pollutant concentration inside the street canyon is related to the number and intensity of vortices. In the case of windward side heating, the residue concentration ratio was the largest when AR = 1. When the aspect ratio increased from 1.0 to 3.5, the residue concentration ratio gradually decreased. In contrast to the leeward side or ground heating scenarios, the aspect ratio has a minor impact on the residue concentration ratio [147]. However, simple expressions linking the flow regimes with aspect ratios and Ri numbers are lacking. If geometric shapes or wind conditions change, labor-intensive processing and computationally intensive calculations are still needed, and most studies were conducted at symmetric street canyons, but due to the different geometries of the buildings, an asymmetric street canyon is more common in urban areas.

#### 3.2.5. Other Factors

Recent studies under non-isothermal conditions in street canyons have focused on the effects of approaching wind, thermal position, thermal intensity and street canyon geometry. These effects have been discussed above, and some additional factors have also been discussed in other studies. Qu et al. [171] described various thermal exchange models on the flow field in different thermal conditions. Fellini et al. [153] evaluated how the wall roughness influences pollutant dispersion within an idealized street canyon in a wind tunnel. Asphalt and concrete were used as representatives of ground surface materials to simulate the surface albedo influence on the energy balance and urban external wall temperature [143].

## 4. Conclusions

Deteriorating outdoor and indoor air quality has a major effect on human health and can cause enormous economic losses. Therefore, it is imperative and urgent to improve air quality. Understanding the coupling effect of wind and buoyancy on the airflow field and pollutant dispersion is significant for accurately predicting the urban heat island and pollutant prediction under realistic conditions. This paper reviews most of the related studies and categorizes the influential factors on airflow and pollutant dispersion based on two typical built environments. The following conclusions can be drawn:

1. Aimed at the isolated building, the three factors, i.e., approach wind (Section 3.1.1), thermal intensity (Section 3.1.2) and source location (Section 3.1.3), have significant influences on the flow field and pollutant dispersion routes both in and around the isolated building and should not be ignored by residents and architects. In the context of the global airborne disease pandemic, reasonable prevention and control measures based on the coupled effects of wind and thermal force are appropriate methods to prevent or reduce exposure. However, research gaps in the literature have been identified. Multiple studies strongly rely on costly experiments and numerical simulations, while additional works are required once the parameters change. Further study linking the environmental indicators to the influencing factor and Ri number is needed.
2. Aimed at the street canyon, the four parameters, i.e., approaching wind (Section 3.2.1), thermal position (Section 3.2.2), thermal intensity (Section 3.2.3) and canyon geometry (Section 3.2.4), played major roles in the airflow and pollutant concentration decisions in urban areas. The flow regimes in street canyons with various aspect ratios, thermal positions and intensities varied in different studies. We suggest further simulations using the wind tunnel experiments database with a similar aspect ratio, thermal position and Ri number to validate the numerical methods. In addition, the accuracy of the Reynolds number-independent criterion under non-isothermal conditions remains questionable, and it is worth exploring and investigating whether the results obtained from scaled models can be generalized to all full-scale models in follow-up investigations.

In summary, this paper systematically articulates the approaches and research outcomes under the combined effect of wind and buoyancy from the street canyon scale to an isolated building and reviews the effects of different influential factors on pollutant dispersion. However, the existing studies of the relevant factors are largely independent of each other in two typical built environments. The real gap in our knowledge is how the complexities of the real world affect the behavior of flow and dispersion of contaminants, and it is more important to study the impact of a combination of influencing factors to identify which factors are more pronounced. By adopting the orthogonal experiment design [172,173] to analyse the order of magnitude of various influencing factors under the combined effect of wind and buoyancy, the effects of various influencing factors under a typical built environment can be qualified and analysed, subsequently obtaining a better understanding of the flow behavior and pollutant dispersion in complex reality. This study is especially relevant for policy makers and urban planners for designing healthier and comfortable indoor and outdoor environments, and continued partnerships with experts from other sectors of practice and industry are of major importance. Collaboration with civil engineers, meteorologists and landscape engineering is key to innovation. This could help to further refine the guidelines, standards and urban development planning.

## Figures and Tables

**Table 1:** Studies on approaching wind speed under non-isothermal conditions around an isolated building.

Ref.	Methods ^a^	Turbulence Model ^b^	Building Geometry	Wind Speeds (m/s)	Ri Number	Data Availability
[97]	CFD	RNG k-ε model	A four-story building (H = 10.8 m)	0.5–4.0	0.11–7.23	Air change rate, distributions of mass fraction of tracer gas
[98]	CFD	SST k-ω model	A ten-story building (H = 30 m)	1.0–13.8	0–14.0	Reentry ratios from the source unit to the other units
[99]	CFD	RNG k-ε model	A twenty-story building (H = 58 m)	0.4–6.4	0–156.9	Concentration distributions, reentry ratios from the source unit to the other units
[100]	WT + CFD	Standard k-ε model	1:40 scaled model (H = 0.3 m)	1.0–9.0	0–2.33	Velocity contours, concentration distributions
[101]	CFD	SST k-ω model	Cubic (H = 4.0 m)	1.0–3.0	0–1.61	Velocity distributions, temperature distributions
[110]	CFD	Baseline k-ω model	H = 3.2 m	1.0–5.0	0.1–2.50	Average concentrations, reentry ratios

**^a^** CFD refers to computational fluid dynamics; WT refers to wind tunnel measurements; ^b^ RNG refers to the renormalization group; SST refers to shear stress transport.

**Table 2:** Studies on thermal intensity under non-isothermal conditions around an isolated building.

Ref.	Research Methods ^a^	Turbulence Models ^b^	Building Geometry	Heated Surface ^c^	Heated Intensity (*T_W_* – *T_ref_*)	Ri Number	Data Availability
[98]	CFD	SST k-ω model + enhanced wall function	H = 30 m	WH/LH	0–15 K	0–14	Reentry ratios
[99]	CFD	RNG k-ε model + enhanced wall function	H = 58 m	WH/LH	0–13 K	0–156.9	Concentration distributions, air exchange rate, reentry ratios
[100]	WT + CFD	Standard k-ε model	H = 0.3 m	LH + GH	0–240 K	0–2.33	Velocity distributions, concentration distributions
[112]	WT	-	H = 0.2 m	RH	0–250 K	0–1.15	Temperature distributions, concentration distributions
[114]	CFD	RNG k-ε model + standard wall function	H = 0.9 m	WH/LH	0–15 K	0–0.027	Vortex core locations, pollutant concentrations
[115]	CFD	LES (Vortex Method)	H = 0.16 m	GH	0–114.81 K	0–1.5	Velocity distributions, temperature distributions, concentration distributions
[116]	CFD	URANS SST k-ω + IDDES SST k-ω	H = 0.16 m	GH	0/33.6 K	0/0.085	Velocity distributions, concentration distributions
[117]	WT + CFD	RNG k-ε model	H = 0.15 m	GH	3–58 K	0.057–1.13	Velocity distributions, temperature distributions, concentration distributions
[118]	WT	-	H = 0.19 m	LH	0–152 K	0–1.6	Velocity distributions, turbulent kinetic energy distributions, temperature distributions
[119]	CFD	Standard k-ε model	H = 10 m	AH	5–50 K	6.81–68.06	Recirculation region

^a^ CFD refers to computational fluid dynamics; WT refers to wind tunnel measurements; ^b^ RNG refers to the renormalization group; SST refers to shear stress transport; URANS refers to unsteady Reynolds-averaged Navier–Stokes; LES refers to large eddy simulation; IDDES refers to improved delayed detached eddy simulation; ^c^ NH refers to no surface heating; LH refers to leeward surface heating; WH refers to windward surface heating; RH refers to rooftop heating; and AH refers to all building surfaces heating.

**Table 3:** Studies on thermal position under non-isothermal conditions in street canyons.

Ref.	Research Method ^a^	Street Canyon Dimension ^d^	Aspect Ratio	Heated Surface ^c^	Source Category	Ri Number	Data Availability
[65]	CFD	2D	1	NH/GH/WH/LH	Line source	4.57	Streamline field, concentration distributions
[130]	CFD	2D	1	NH/GH/WH/LH	CO; line source	1.1~39.04	Airflow characteristics, concentration distributions
[132]	CFD	3D	0.75	GH/WH/LH	N. A	N.A	Pressure distributions, air exchange rates
[134]	CFD	2D	1	NH/GH/WH/LH	CO; line source	N.A	Airflow characteristics, concentration distributions, vertical velocity
[144]	CFD	2D	1	NH/GH/WH/LH/AH	CO; particle/line source	2.63~5.26	Streamline and velocity fields, concentration distributions, particle distributions
[145]	CFD	2D	0.5/0.67/1/2/3	NH/GH/WH/LH/AH	CO; line source	0~4.0	Velocity distributions, concentration distributions
[146]	CFD	3D	1	NH/GH/WH/LH	Line source	0.013/0.173	Velocity profiles, pollutant concentrations
[147]	CFD	2D	1/2/3.5	NH/GH/WH/LH	Point source	0~3.75	Streamline field, temperature distributions, concentration distributions
[149]	CFD	2D	1.12	NH/GH/WH/LH	N. A	0/2.68	Concentration distributions
[150]	CFD	2D	0.1/0.5/1/2	AH/GH+LH/GH+WH/GH	CO; line source	6.6	Flow field, temperature distributions, concentration distributions
[151]	CFD	3D	1	NH/GH/WH/LH	N. A	0~2.7	Streamline field, turbulent momentum fluxes
[152]	CFD	3D	1	WH/LH	N. A	0~2.14	Turbulent intensity distributions, temperature distributions
[153]	WT	2D	1/1.5	NH/WH/LH	Ethane; line source	0~10.41	Velocity profiles, turbulent kinetic energy fields

^a^ CFD refers to computational fluid dynamics; WT refers to wind tunnel measurements; ^c^ NH refers to no surface heating; LH refers to leeward surface heating; WH refers to windward surface heating; RH refers to rooftop heating; AH refers to all building surfaces heating; ^d^ 2D refers to two-dimensional; 3D refers to three-dimensional.

**Table 4:** Studies on thermal intensity under non-isothermal conditions in street canyons.

Ref.	Research Method ^a^	Street Canyon Dimension ^d^	Aspect Ratio	Heated Surface ^c^	Heated Intensity (*T_W_* − *T_ref_*)	*Ri* Number	Data Availability
[65]	CFD	2D	1	WH	2–15 K	0.91–6.86	Streamline fields, concentration distributions
[73]	CFD	2D	0.6–3.6	GH	0–16 K	0–12.33	Streamline fields, temperature distributions
[130]	CFD	2D	1	WH	2–15 K	1.96–14.6	Airflows profiles, concentration distributions, velocity distributions
[131]	CFD	3D	1	WH/LH/AH	47–107 K	0.058–1.54	Trajectories of the center of the main vortex, Velocity profiles, temperature distributions
[135]	CFD	3D	1	GH	0–10 K	0–34.0	Velocity distributions, turbulent kinetic energy distributions
[136]	CFD	2D	1	WH + GH	WH: 0~20 K; GH: 0~30 K	0~1.611	Velocity distributions, temperature distributions
[141]	WT	3D	0.8	AH	0~107 K	0~1.09	Velocity distributions, turbulent kinetic energy distributions, temperature distributions
[152]	CFD	3D	1	WH/LH	0~15 K	0~2.14	Turbulent kinetic energy distributions
[153]	WT	2D	1.0/1.5	WH/LH	0~240 K	0~10.41	Velocity profiles, turbulent kinetic energy field
[157]	CFD	3D	1	GH	N. A	0~2.4	Mean flow distributions, velocity variance, temperature distributions, concentration distributions

^a^ CFD refers to computational fluid dynamics; WT refers to wind tunnel measurements; ^c^ NH refers to no surface heating; LH refers to leeward surface heating; WH refers to windward surface heating, RH refers to rooftop heating; AH refers to all building surfaces heating; ^d^ 2D refers to two-dimensional; 3D refers to three-dimensional.

## Data Availability

The raw data supporting the conclusions of this article will be made available by the authors, without undue reservation.

## References

[1] Chan C.K., Yao X.H. (2008). Air pollution in mega cities in China. Atmos. Environ..

[2] Hachem M., Saleh N., Paunescu A.C., Momas I., Bensefa-Colas L. (2019). Exposure to traffic air pollutants in taxicabs and acute adverse respiratory effects: A systematic review. Sci. Total Environ..

[3] Zheng S., Wang J., Sun C., Zhang X., Kahn M.E. (2019). Air pollution lowers Chinese urbanites’ expressed happiness on social media. Nat. Hum. Behav..

[4] Buccolieri R., Hang J. (2019). Recent advances in urban ventilation assessment and flow modelling. Atmosphere.

[5] Lee S.C., Chang M. (2000). Indoor and outdoor air quality investigation at schools in Hong Kong. Chemosphere.

[6] Khreis H., Nieuwenhuijsen M.J. (2017). Traffic-related air pollution and childhood asthma: Recent advances and remaining gaps in the exposure assessment methods. Int. J. Environ. Res. Public Health.

[7] Neidell M.J. (2004). Air pollution, health, and socio-economic status: The effect of outdoor air quality on childhood asthma. J. Health Econ..

[8] Jovanović M., Vučićević B., Turanjanin V., Živković M., Spasojević V. (2014). Investigation of indoor and outdoor air quality of the classrooms at a school in Serbia. Energy.

[9] Wyon D.P. (2004). The effects of indoor air quality on performance and productivity. Indoor Air..

[10] Hassan A.M., EI Mokadem A.A.F., Megahed N.A., Eleinen O.M.A. (2020). Improving outdoor air quality based on building morphology: Numerical investigation. Front. Archit. Res..

[11] Mannan M., Al-Ghamdi S.G. (2021). Indoor air quality in buildings: A comprehensive review on the factors influencing air pollution in residential and commercial structure. Int. J. Environ. Res. Public Health.

[12] Zhou C., Li S., Wang S. (2018). Examining the impacts of urban form on air pollution in developing countries: A case study of China’s megacities. Int. J. Environ. Res. Public Health.

[13] Yang W., Li L. (2018). Efficiency evaluation of industrial waste gas control in China: A study based on data envelopment analysis (DEA) model. J. Clean Prod..

[14] Cong X. (2018). Air pollution from industrial waste gas emissions is associated with cancer incidences in Shanghai, China. Environ. Sci. Pollut. Res..

[15] Seifert A.H., Rittmann S., Bernacchi S., Herwig C. (2013). Method for assessing the impact of emission gasses on physiology and productivity in biological methanogenesis. Bioresour. Technol..

[16] Cui D., Li X., Liu J., Yuan L., Mak C.M., Fan Y., Kwok L. (2021). Effects of building layouts and envelope features on wind flow and pollutant exposure in height-asymmetric street canyons. Build. Environ..

[17] Kwak K., Woo S., Kim K., Lee S., Bae G., Ma Y., Sunwoo Y., Baik J. (2018). On-road air quality associated with traffic composition and street-canyon ventilation: Mobile monitoring and CFD modeling. Atmosphere.

[18] Pant P., Harrison R.M. (2013). Estimation of the contribution of road traffic emissions to particulate matter concentrations from field measurements: A review. Atmos. Environ..

[19] Choudhary A., Gokhale S. (2016). Urban real-world driving traffic emissions during interruption and congestion. Transport. Res. Part D-Transport. Environ..

[20] Wu Z., Zhang X., Wu M. (2016). Mitigating construction dust pollution: State of the art and the way forward. J. Clean Prod..

[21] Guo P., Tian W., Li H., Zhang G., Li J. (2020). Global characteristics and trends of research on construction dust: Based on bibliometric and visualized analysis. Environ. Sci. Pollut. Res..

[22] Yan H., Ding G., Feng K., Zhang L., Li H., Wang Y., Wu T. (2020). Systematic evaluation framework and empirical study of the impacts of building construction dust on the surrounding environment. J. Clean Prod..

[23] Cincinelli A., Martellini T. (2017). Indoor air quality and health. Int. J. Environ. Res. Public Health.

[24] Tran V.V., Park D., Lee Y.C. (2020). Indoor air pollution, related human diseases, and recent trends in the control and improvement of indoor air quality. Int. J. Environ. Res. Public Health.

[25] Spengler J.D., Sexton K. (1983). Indoor air pollution: A public health perspective. Science.

[26] Antiochia R. (2020). Nanobiosensors as new diagnostic tools for SARS, MERS and COVID-19: From past to perspectives. Microchim. Acta.

[27] Zhang S., Lin Z. (2021). Dilution-based evaluation of airborne infection risk-Thorough expansion of Wells-Riley model. Build. Environ..

[28] Guo Y., Qian H., Sun Z., Cao J., Liu F., Luo X., Ling R., Weschler L.B., Mo J., Zhang Y. (2021). Assessing and controlling infection risk with Wells-Riley model and spatial flow impact factor (SFIF). Sustain. Cities Soc..

[29] Ravindra K., Goyal A., Mor S. (2021). Does airborne pollen influence COVID-19 outbreak?. Sustain. Cities Soc..

[30] Xiao J., Fang M., Chen Q., He B. (2020). SARS, MERS and COVID-19 among healthcare workers: A narrative review. J. Infect. Public Health.

[31] Guarner J. (2020). Three emerging coronaviruses in two decades: The story of SARS, MERS, and now COVID-19. Am. J. Clin. Pathol..

[32] Li Y.D., Chi W.Y., Su J.H., Ferrall L., Hung C.F., Wu T.C. (2020). Coronavirus vaccine development: From SARS and MERS to COVID-19. J. Biomed. Sci..

[33] Sanchez B., Santiago J.L., Martilli A., Palacios M., Kirchner F. (2016). CFD modeling of reactive pollutant dispersion in simplified urban configurations with different chemical mechanisms. Atmos. Chem. Phys..

[34] Georgakis C., Santamouris M. (2008). On the estimation of wind speed in urban canyons for ventilation purposes—Part 1: Coupling between the undisturbed wind speed and the canyon wind. Build. Environ..

[35] Santamouris M., Papanikolaou N., Koronakis I., Livada I., Asimakopoulos D. (1999). Thermal and air flow characteristics in a deep pedestrian canyon under hot weather conditions. Atmos. Environ..

[36] Nazridoust K., Ahmadi G. (2006). Airflow and pollutant transport in street canyons. J. Wind Eng. Ind. Aerodyn..

[37] Wang Q., Hang J., Fan Y., Li Y. (2020). Urban plume characteristics under various wind speed, heat flux, and stratification conditions. Atmos. Environ..

[38] Moonen P., Dorer V., Carmeliet J. (2012). Effect of flow unsteadiness on the mean wind flow pattern in an idealized urban environment. J. Wind Eng. Ind. Aerodyn..

[39] Zhang H., Xu T., Zong Y., Tang H., Liu X., Wang Y. (2015). Influence of meteorological conditions on pollutant dispersion in street canyon. Procedia Eng..

[40] Yu Y., Kwok K.C.S., Liu X.P., Zhang Y. (2017). Air pollutant dispersion around high-rise buildings under different angles of wind incidence. J. Wind Eng. Ind. Aerodyn..

[41] Huang Y.D., Hou R.W., Liu Z.Y., Song Y., Cui P.Y., Kim C.N. (2019). Effects of wind direction on the airflow and pollutant dispersion inside a long street canyon. Aerosol. Air Qual. Res..

[42] Milliez M., Carissimo B. (2007). Numerical simulations of pollutant dispersion in an idealized urban area, for different meteorological conditions. Bound. Layer Meteor..

[43] Kim J.J., Baik J.J. (2005). Physical experiments to investigate the effects of street bottom heating and inflow turbulence on urban street-canyon flow. Adv. Atmos. Sci..

[44] An K., Fung J.C.H., Yim S.H.L. (2013). Sensitivity of inflow boundary conditions on downstream wind and turbulence profiles through building obstacles using a CFD approach. J. Wind Eng. Ind. Aerodyn..

[45] García-Sánchez C., Van Tendeloo G., Gorlé C. (2017). Quantifying inflow uncertainties in RANS simulations of urban pollutant dispersion. Atmos. Environ..

[46] Tominaga Y., Stathopoulos T. (2013). CFD simulation of near-field pollutant dispersion in the urban environment: A review of current modeling techniques. Atmos. Environ..

[47] Wang L., Pan Q., Zheng X.P., Yang S.S. (2017). Effects of low boundary walls under dynamic inflow on flow field and pollutant dispersion in an idealized street canyon. Atmos. Pollut. Res..

[48] Cheng W.C., Liu C.H. (2011). Large-eddy simulation of turbulent transports in urban street canyons in different thermal stabilities. J. Wind Eng. Ind. Aerodyn..

[49] Shen Z., Cui G., Zhang Z. (2017). Turbulent dispersion of pollutants in urban-type canopies under stable stratification conditions. Atmos. Environ..

[50] Boppana V.B.L., Xie Z.T., Castro I.P. (2014). Thermal stratification effects on flow over a generic urban canopy. Bound. Layer Meteor..

[51] Li X.X., Britter R., Norford L.K. (2016). Effect of stable stratification on dispersion within urban street canyons: A large-eddy simulation. Atmos. Environ..

[52] Sessa V., Xie Z.T., Herring S. (2020). Thermal stratification effects on turbulence and dispersion in internal and external boundary layers. Bound. Layer Meteor..

[53] Liu F., Qian H., Luo Z., Zheng X. (2021). The impact of indoor thermal stratification on the dispersion of human speech droplets. Indoor Air.

[54] Guo D., Zhao P., Wang R., Yao R., Hu J. (2020). Numerical simulations of the flow field and pollutant dispersion in an idealized urban area under different atmospheric stability conditions. Process Saf. Environ. Protect..

[55] Huang X., Gao L., Guo D., Yao R. (2021). Impacts of high-rise building on urban airflows and pollutant dispersion under different temperature stratifications: Numerical investigations. Atmos. Pollut. Res..

[56] Xiong Y., Chen H. (2022). Impacts of uneven surface heating of an ideal street canyon on airflows and indoor ventilation: Numerical study using OpenFOAM coupled with EnergyPlus. Build. Simul..

[57] Niachou K., Livada I., Santamouris M. (2008). Experimental study of temperature and airflow distribution inside an urban street canyon during hot summer weather conditions—Part I: Air and surface temperatures. Build. Environ..

[58] Niachou K., Livada I., Santamouris M. (2008). Experimental study of temperature and airflow distribution inside an urban street canyon during hot summer weather conditions. Part II: Airflow analysis. Build. Environ..

[59] Xiong Y., Chen H. (2021). Effects of sunshields on vehicular pollutant dispersion and indoor air quality: Comparison between isothermal and nonisothermal conditions. Build. Environ..

[60] Louka P., Vachon G., Sini J.F., Mestayer P.G., Rosant J.M. (2002). Thermal effects on the airflow in a street canyon–Nantes’99 experimental results and model simulations. Water Air Soil Pollut. Focus.

[61] Britter R.E., Hanna S.R. (2003). Flow and dispersion in urban areas. Annu. Rev. Fluid Mech..

[62] Wang W., Wang X., Ng E. (2021). The coupled effect of mechanical and thermal conditions on pedestrian-level ventilation in high-rise urban scenarios. Build. Environ..

[63] An K., Wong S.M., Fung J.C.H. (2019). Exploration of sustainable building morphologies for effective passive pollutant dispersion within compact urban environments. Build. Environ..

[64] Zhang W.C., Luo X.Y., Peng X.R., Liu R.Z., Jing Y., Zhao F.Y. (2021). Green Roof on the Ventilation and Pollutant Dispersion in Urban Street Canyons under Unstable Thermal Stratification: Aiding and Opposing Effects. Sustain. Cities Soc..

[65] Wang P., Zhao D., Wang W., Mu H., Cai G., Liao C. (2011). Thermal effect on pollutant dispersion in an urban street canyon. Int. J. Environ. Res..

[66] ASHRAE (2015). ASHRAE Handbook.

[67] Li X.X., Liu C.H., Leung D.Y.C. (2009). Numerical investigation of pollutant transport characteristics inside deep urban street canyons. Atmos. Environ..

[68] He L., Hang J., Wang X., Lin B., Li X., Lan G. (2017). Numerical investigations of flow and passive pollutant exposure in high-rise deep street canyons with various street aspect ratios and viaduct settings. Sci. Total Environ..

[69] Tong N.Y., Leung D.Y. (2012). Effects of building aspect ratio, diurnal heating scenario, and wind speed on reactive pollutant dispersion in urban street canyons. J. Environ. Sci..

[70] Liu H., Liang B., Zhu F., Zhang B., Sang J. (2003). A laboratory model for the flow in urban street canyons induced by bottom heating. Adv. Atmos. Sci..

[71] Schrijvers P.J.C., Jonker H.J.J., de Roode S.R., Kenjereš S. (2020). On the daytime micro-climatic conditions inside an idealized 2D urban canyon. Build. Environ..

[72] Santiago J.L., Krayenhoff E.S., Martilli A. (2014). Flow simulations for simplified urban configurations with microscale distributions of surface thermal forcing. Urban Clim..

[73] Kim J.J., Baik J.J. (2001). Urban street-canyon flows with bottom heating. Atmos. Environ..

[74] Larsen T., Heiselberg P. (2008). Single-sided natural ventilation driven by wind pressure and temperature difference. Energy Build..

[75] Ruck B. (1993). Wind-tunnel measurements of flow field characteristics around a heated model building. J. Wind Eng. Ind. Aerodyn..

[76] Shu C., Wang L.L., Mortezazadeh M. (2020). Dimensional analysis of Reynolds independence and regional critical Reynolds numbers for urban aerodynamics. J. Wind Eng. Ind. Aerodyn..

[77] Castro I.P., Robins A.G. (1977). The flow around a surface-mounted cube in uniform and turbulent streams. J. Fluid Mech..

[78] Hoydysh W.G. A scale model study of the dispersion of pollution in street canyons. Proceedings of the 67th Annual Meeting of the Air Pollution Control Association.

[79] Snyder W.H. (1972). Similarity criteria for the application of fluid models to the study of air pollution meteorology. Bound. Layer Meteor..

[80] Li Q., Liang J., Wang Q., Chen Y., Yang H., Ling H., Luo Z., Hang J. (2022). Numerical investigations of urban pollutant dispersion and building intake fraction with various 3D building configurations and tree plantings. Int. J. Environ. Res. Public Health.

[81] Chew L.W., Aliabadi A.A., Norford L.K. (2018). Flows across high aspect ratio street canyons: Reynolds number independence revisited. Environ. Fluid Mech..

[82] Yang H., Lam C.K.C., Lin Y., Chen L., Mattsson M., Sandberg M., Hayati A., Claesson L., Hang J. (2021). Numerical investigations of Re-independence and influence of wall heating on flow characteristics and ventilation in full-scale 2D street canyons. Build. Environ..

[83] Hang J., Lin M., Wong D.C., Wang X., Wang B., Buccolieri R. (2016). On the influence of viaduct and ground heating on pollutant dispersion in 2D street canyons and toward single-sided ventilated buildings. Atmos. Pollut. Res..

[84] Pramanik S., Achari A.M., Das M.K. (2012). Numerical simulation of a turbulent confined slot impinging jet. Ind. Eng. Chem. Res..

[85] Li Z., Zhang H., Wen C.Y., Yang A.S., Juan Y.H. (2020). Effects of height-asymmetric street canyon configurations on outdoor air temperature and air quality. Build. Environ..

[86] Zhao Y., Li H., Kubilay A., Carmeliet J. (2021). Buoyancy effects on the flows around flat and steep street canyons in simplified urban settings subject to a neutral approaching boundary layer: Wind tunnel PIV measurements. Sci. Total Environ..

[87] Nazarian N., Kleissl J. (2016). Realistic solar heating in urban areas: Air exchange and street-canyon ventilation. Build. Environ..

[88] Memon R.A., Leung D.Y.C. (2011). On the heating environment in street canyon. Environ. Fluid Mech..

[89] Hu T., Yoshie R. (2020). Effect of atmospheric stability on air pollutant concentration and its generalization for real and idealized urban block models based on field observation data and wind tunnel experiments. J. Wind Eng. Ind. Aerodyn..

[90] Tan Z., Dong J., Xiao Y., Tu J. (2015). A numerical study of diurnally varying surface temperature on flow patterns and pollutant dispersion in street canyons. Atmos. Environ..

[91] Nazarian N., Martilli A., Kleissl J. (2018). Impacts of realistic urban heating, part I: Spatial variability of mean flow, turbulent exchange and pollutant dispersion. Bound. Layer Meteorol..

[92] Freire L.S., Chamecki M., Bou-Zeid E., Dias N.L. (2019). Critical flux Richardson number for Kolmogorov turbulence enabled by TKE transport. Q. J. R. Meteorol. Soc..

[93] Zhao Y., Chew L.W., Kubilay A., Carmeliet J. (2020). Isothermal and non-isothermal flow in street canyons: A review from theoretical, experimental and numerical perspectives. Build. Environ..

[94] Nakamura Y., Oke T.R. (1988). Wind, temperature and stability conditions in an east-west oriented urban canyon. Atmos. Environ..

[95] Uehara K., Murakami S., Oikawa S., Wakamatsu S. (2000). Wind tunnel experiments on how thermal stratification affects flow in and above urban street canyons. Atmos. Environ..

[96] Cheng X.L., Zeng Q.C., Hu F. (2011). Characteristics of gusty wind disturbances and turbulent fluctuations in windy atmospheric boundary layer behind cold fronts. J. Geophys. Res. Atmos..

[97] Gao N.P., Niu J.L., Perino M., Heiselberg P. (2008). The airborne transmission of infection between flats in high-rise residential buildings: Tracer gas simulation. Build. Environ..

[98] Liu X., Wu M., An Z., Chen T. (2022). Study on the combined effect of wind and buoyancy on cross-unit contamination around a high-rise residential building. Sustain. Cities Soc..

[99] Mu D., Gao N., Zhu T. (2018). CFD investigation on the effects of wind and thermal wall-flow on pollutant transmission in a high-rise building. Build. Environ..

[100] Huang Y.D., Xu N., Ren S.Q., Qian L.B., Cui P.Y. (2021). Numerical investigation of the thermal effect on flow and dispersion of rooftop stack emissions with wind tunnel experimental validations. Environ. Sci. Pollut. Res..

[101] Park J., Sun X., Choi J.I., Rhee G.H. (2017). Effect of wind and buoyancy interaction on single-sided ventilation in a building. J. Wind Eng. Ind. Aerodyn..

[102] Blocken B. (2015). Computational Fluid Dynamics for urban physics: Importance, scales, possibilities, limitations and ten tips and tricks towards accurate and reliable simulations. Build. Environ..

[103] Santiago J.L., Dejoan A., Martilli A., Martin F., Pinelli A. (2010). Comparison between large-eddy simulation and Reynolds-averaged Navier–Stokes computations for the MUST field experiment. Part I: Study of the flow for an incident wind directed perpendicularly to the front array of containers. Bound. Layer Meteor..

[104] Dejoan A., Santiago J.L., Martilli A., Martin F., Pinelli A. (2010). Comparison between large-eddy simulation and Reynolds-averaged Navier–Stokes computations for the MUST field experiment. Part II: Effects of incident wind angle deviation on the mean flow and plume dispersion. Bound.-Layer Meteor..

[105] Tominaga Y., Stathopoulos T. (2012). CFD modeling of pollution dispersion in building array: Evaluation of turbulent scalar flux modeling in RANS model using LES results. J. Wind Eng. Ind. Aerodyn..

[106] Du Y., Blocken B., Pirker S. (2020). A novel approach to simulate pollutant dispersion in the built environment: Transport-based recurrence CFD. Build. Environ..

[107] Gronemeier T., Raasch S., Ng E. (2017). Effects of unstable stratification on ventilation in Hong Kong. Atmosphere.

[108] Gousseau P., Blocken B., Van Heijst G.J.F. (2011). CFD simulation of pollutant dispersion around isolated buildings: On the role of convective and turbulent mass fluxes in the prediction accuracy. J. Hazard. Mater..

[109] Salim S.M., Buccolieri R., Chan A., Di Sabatino S. (2011). Numerical simulation of atmospheric pollutant dispersion in an urban street canyon: Comparison between RANS and LES. J. Wind Eng. Ind. Aerodyn..

[110] Wang J., Huo Q., Zhang T., Wang S., Battaglia F. (2020). Numerical investigation of gaseous pollutant cross-transmission for single-sided natural ventilation driven by buoyancy and wind. Build. Environ..

[111] Mu D., Shu C., Gao N., Zhu T. (2017). Wind tunnel tests of inter-flat pollutant transmission characteristics in a rectangular multi-storey residential building, part B: Effect of source location. Build. Environ..

[112] Huang X.T., Huang Y.D., Xu N., Luo Y., Cui P.Y. (2020). Thermal effects on the dispersion of rooftop stack emission in the wake of a tall building within suburban areas by wind-tunnel experiments. J. Wind Eng. Ind. Aerodyn..

[113] Yassin M.F., Alhajeri N.S., Elmi A.A., Malek M.J., Shalash M. (2021). Numerical simulation of gas dispersion from rooftop stacks on buildings in urban environments under changes in atmospheric thermal stability. Environ. Monit. Assess..

[114] Liu X., Wu X., Wu M., Shi C. (2020). The impact of building surface temperature rise on airflow and cross-contamination around high-rise building. Environ. Sci. Pollut. Res..

[115] Zhou X., Ying A., Cong B., Kikumoto H., Ooka R., Kang L., Hu H. (2021). Large eddy simulation of the effect of unstable thermal stratification on airflow and pollutant dispersion around a rectangular building. J. Wind Eng. Ind. Aerodyn..

[116] Valger S. (2022). Estimation of pollutant dispersion around a building within non-isothermal boundary layer using detached eddy simulation. Therm. Sci..

[117] Guo D., Yang F., Shi X., Li Y., Yao R. (2021). Numerical simulation and wind tunnel experiments on the effect of a cubic building on the flow and pollutant diffusion under stable stratification. Build. Environ..

[118] Richards K., Schatzmann M., Leitl B. (2006). Wind tunnel experiments modelling the thermal effects within the vicinity of a single block building with leeward wall heating. J. Wind Eng. Ind. Aerodyn..

[119] Nardecchia F., Gugliermetti F., Bisegna F. (2016). How temperature affects the airflow around a single-block isolated building. Energy Build..

[120] Yassin M.F. (2013). A wind tunnel study on the effect of thermal stability on flow and dispersion of rooftop stack emissions in the near wake of a building. Atmos. Environ..

[121] Pavlidis C.L., Palampigik A.V., Vasilopoulos K., Lekakis I.C., Sarris I.E. (2022). Air Flow Study Around Isolated Cubical Building in the City of Athens under Various Climate Conditions. Appl. Sci..

[122] Masoumi-Verki S., Gholamalipour P., Haghighat F., Eicker U. (2021). Embedded LES of thermal stratification effects on the airflow and concentration fields around an isolated high-rise building: Spectral and POD analyses. Build. Environ..

[123] Bazdidi-Tehrani F., Gholamalipour P., Kiamansouri M., Jadidi M. (2019). Large eddy simulation of thermal stratification effect on convective and turbulent diffusion fluxes concerning gaseous pollutant dispersion around a high-rise model building. J. Build. Eng..

[124] Snyder W.H. (1981). Guideline for Fluid Modeling of Atmospheric Diffusion.

[125] Chew L.W., Glicksman L.R., Norford L.K. (2018). Buoyant flows in street canyons: Comparison of RANS and LES at reduced and full scales. Build. Environ..

[126] Wang J., Wang S., Zhang T., Battaglia F. (2017). Assessment of single-sided natural ventilation driven by buoyancy forces through variable window configurations. Energy Build..

[127] Li Z., Ming T., Liu S., Peng C., de Richter R., Li W., Wen C.Y. (2021). Review on pollutant dispersion in urban areas—Part A: Effects of mechanical factors and urban morphology. Build. Environ..

[128] Cui P.Y., Li Z., Tao W.Q. (2014). Investigation of Re-independence of turbulent flow and pollutant dispersion in urban street canyon using numerical wind tunnel (NWT) models. Int. J. Heat Mass Tran..

[129] Wise D.J., Boppana V.B.L., Li K.W., Poh H.J. (2018). Effects of minor changes in the mean inlet wind direction on urban flow simulations. Sustain. Cities Soc..

[130] Xie X., Huang Z., Wang J., Xie Z. (2005). Thermal effects on vehicle emission dispersion in an urban street canyon. Transp. Res. Part D-Transp. Environ..

[131] Allegrini J., Dorer V., Carmeliet J. (2013). Wind tunnel measurements of buoyant flows in street canyons. Build. Environ..

[132] Wang Y., Zhong K., Zhang N., Kang Y. (2014). Numerical analysis of solar radiation effects on flow patterns in street canyons. Eng. Appl. Comput. Fluid Mech..

[133] Cui P.Y., Li Z., Tao W.Q. (2016). Wind-tunnel measurements for thermal effects on the air flow and pollutant dispersion through different scale urban areas. Build. Environ..

[134] Xie X., Huang Z., Wang J., Xie Z. (2005). The impact of solar radiation and street layout on pollutant dispersion in street canyon. Build. Environ..

[135] Xie X., Liu C.H., Leung D.Y.C., Leung M.K. (2006). Characteristics of air exchange in a street canyon with ground heating. Atmos. Environ..

[136] Battista G., Mauri L. (2016). Numerical study of buoyant flows in street canyon caused by ground and building heating. Energy Procedia.

[137] Allegrini J. (2018). A wind tunnel study on three-dimensional buoyant flows in street canyons with different roof shapes and building lengths. Build. Environ..

[138] Lin Y., Ichinose T., Yamao Y., Mouri H. (2020). Wind velocity and temperature fields under different surface heating conditions in a street canyon in wind tunnel experiments. Build. Environ..

[139] Kovar-Panskus A., Moulinneuf L., Savory E., Abdelqari A., Sini J.F., Rosant J.M., Robins A., Toy N. (2002). A wind tunnel investigation of the influence of solar-induced wall-heating on the flow regime within a simulated urban street canyon. Water Air Soil Pollut. Focus.

[140] Huang X., Wang H., Gao L. (2022). Numerical simulation of airflow and dispersion in 3D street canyons: The effect of atmospheric temperature stratification. Environ. Technol..

[141] Tsalicoglou C., Allegrini J., Carmeliet J. (2020). Non-isothermal flow between heated building models. J. Wind Eng. Ind. Aerodyn..

[142] Allegrini J., Dorer V., Defraeye T., Carmeliet J. (2012). An adaptive temperature wall function for mixed convective flows at exterior surfaces of buildings in street canyons. Build. Environ..

[143] Nazarian N., Kleissl J. (2015). CFD simulation of an idealized urban environment: Thermal effects of geometrical characteristics and surface materials. Urban Clim..

[144] Lin L., Hang J., Wang X., Wang X., Fan S., Fan Q., Liu Y. (2016). Integrated effects of street layouts and wall heating on vehicular pollutant dispersion and their reentry toward downstream canyons. Aerosol. Air Qual. Res..

[145] Hang J., Chen X., Chen G., Chen T., Lin Y., Luo Z., Zhang X., Wang Q. (2020). The influence of aspect ratios and wall heating conditions on flow and passive pollutant exposure in 2D typical street canyons. Build. Environ..

[146] Witri A.M.Y., Che Sidik N.A., Mohamed Salim S. (2014). Numerical simulation of wind flow structures and pollutant dispersion within street canyon under thermally unstable atmospheric conditions. Applied Mechanics and Materials.

[147] Kim J.J., Baik J.J. (1999). A numerical study of thermal effects on flow and pollutant dispersion in urban street canyons. J. Appl. Meteorol. Climatol..

[148] Mei S.J., Hu J.T., Liu D., Zhao F.Y., Li Y., Wang Y., Wang H.Q. (2017). Wind driven natural ventilation in the idealized building block arrays with multiple urban morphologies and unique package building density. Energy Build..

[149] Sini J.F., Anquetin S., Mestayer P.G. (1996). Pollutant dispersion and thermal effects in urban street canyons. Atmos. Environ..

[150] Xie X., Liu C.H., Leung D.Y.C. (2007). Impact of building facades and ground heating on wind flow and pollutant transport in street canyons. Atmos. Environ..

[151] Park S.B., Baik J.J., Raasch S., Letzel M.O. (2012). A large-eddy simulation study of thermal effects on turbulent flow and dispersion in and above a street canyon. J. Appl. Meteorol. Climatol..

[152] Cai X.M. (2012). Effects of wall heating on flow characteristics in a street canyon. Bound. Layer Meteorol..

[153] Fellini S., Ridolfi L., Salizzoni P. (2020). Street canyon ventilation: Combined effect of cross-section geometry and wall heating. Q. J. R. Meteorol. Soc..

[154] Kanda I., Yamao Y. (2016). Passive scalar diffusion in and above urban-like roughness under weakly stable and unstable thermal stratification conditions. J. Wind Eng. Ind. Aerodyn..

[155] Cai J., Chen J., Cheng H., Zi S., Xiao J., Xia F., Zhao J. (2022). The effects of thermal stratification on airborne transport within the urban roughness sublayer. Int. J. Heat Mass Transfer..

[156] Shi X., Guo D., Yao R. (2021). Numerical simulation studies of the flow field and pollutant dispersion in the street canyon under different thermal stratifications. IOP Conf. Ser. Earth Environ. Sci..

[157] Li X.X., Britter R.E., Koh T.Y., Norford L.K., Liu C.H., Entekhabi D., Leung D.Y. (2010). Large-eddy simulation of flow and pollutant transport in urban street canyons with ground heating. Bound. Layer Meteorol..

[158] Hang J., Luo Z., Wang X., He L., Wang B., Zhu W. (2017). The influence of street layouts and viaduct settings on daily CO exposure and intake fraction in idealized urban canyons. Environ. Pollut..

[159] Zhang K., Chen G., Wang X., Liu S., Mak C.M., Fan Y., Hang J. (2019). Numerical evaluations of urban design technique to reduce vehicular personal intake fraction in deep street canyons. Sci. Total Environ..

[160] Vardoulakis S., Fisher B.E., Pericleous K., Gonzalez-Flesca N. (2003). Modeling air quality in street canyons: A review. Atmos. Environ..

[161] Li X.X., Liu C.H., Leung D.Y., Lam K.M. (2006). Recent progress in CFD modelling of wind field and pollutant transport in street canyons. Atmos. Environ..

[162] Fernando H.J., Zajic D., Di Sabatino S., Dimitrova R., Hedquist B., Dallman A. (2010). Flow, turbulence, and pollutant dispersion in urban atmospheres. Phys. Fluid..

[163] Zhang Y., Gu Z., Yu C.W. (2018). Review on numerical simulation of airflow and pollutant dispersion in urban street canyons under natural background wind conditions. Aerosol. Air. Qual. Res..

[164] Oke T.R. (1988). Street design and urban canopy layer climate. Sci. Total Environ..

[165] Meroney R.N., Pavegeau M., Rafailidis S., Schatzmann M. (1996). Study of line source characteristics for 2-D physical modelling of pollutant dispersion in street canyons. J. Wind Eng. Ind. Aerodyn..

[166] Memon R.A., Leung D.Y.C., Liu C.H. (2010). Effects of building aspect ratio and wind speed on air temperatures in urban-like street canyons. Build. Environ..

[167] Dallman A., Magnusson S., Britter R., Norford L., Entekhabi D., Fernando H.J.S. (2014). Conditions for thermal circulation in urban street canyons. Build. Environ..

[168] Allegrini J., Dorer V., Carmeliet J. (2014). Buoyant flows in street canyons: Validation of CFD simulations with wind tunnel measurements. Build. Environ..

[169] Xie X., Huang Z., Wang J. (2006). The impact of urban street layout on local atmospheric environment. Build. Environ..

[170] Zhang Y., Gu Z., Lee S.C., Fu T.M., Ho K.F. (2011). Numerical simulation and in situ investigation of fine particle dispersion in an actual deep street canyon in Hong Kong. Indoor Built Environ..

[171] Qu Y., Milliez M., Musson-Genon L., Carissimo B. (2012). Numerical study of the thermal effects of buildings on low-speed airflow taking into account 3D atmospheric radiation in urban canopy. J. Wind Eng. Ind. Aerodyn..

[172] Tang J., Gong G., Su H., Wu F., Herman C. (2016). Performance evaluation of a novel method of frost prevention and retardation for air source heat pumps using the orthogonal experiment design method. Appl. Energy.

[173] Cai J., Chen J., Ahmad S., Zhao J., Cheng H., Zi S., Xiao J. (2020). Investigation into the effect of upstream obstacles and hazardous sources on dispersion in the urban environment with LES model. J. Hazard. Mater..

